# An Unusual Case of Enteropathy-associated T-cell Lymphoma Type 2 with Pulmonary Metastasis

**DOI:** 10.7759/cureus.5714

**Published:** 2019-09-20

**Authors:** Vineel Bhatlapenumarthi, Anannya Patwari, Ahmad D Siddiqui

**Affiliations:** 1 Internal Medicine, Eastern Maine Medical Center, Bangor, USA; 2 Hematology and Oncology, St. Vincent Hospital, Worcester, USA

**Keywords:** lymphoma, pulmonary metastasis, bowel perforation, enteropathy-associated t-cell lymphoma (eatl) type 2

## Abstract

Herein, we present a very rare case of enteropathy-associated T-cell lymphoma (EATL) type 2 with pulmonary metastasis which was biopsy-proven. This is a very rare type of lymphoma, and no case reports or studies of enteropathy-associated T-cell lymphoma with pulmonary metastatic disease were found in the literature review.

This 64-year-old male, who presented with an acute abdomen, was found to have a perforation. Subsequent pathology of the resected specimen showed neoplastic cells consistent with EATL type 2. Four months post-diagnosis, the patient developed shortness of breath. Positron emission tomography (PET) scan revealed multiple metabolically active pulmonary nodules. A biopsy of the nodules was consistent with metastatic EATL type 2 involving the lungs.

## Introduction

Enteropathy-associated T-cell lymphoma (EATL) is a rare peripheral non-Hodgkin's T-cell lymphoma originating from intraepithelial T lymphocytes of the intestines [1]. In general, this condition has a poor prognosis. A common initial presentation of this cancer is an acute abdomen secondary to a small intestinal perforation, necessitating emergency surgery. The diagnosis is rarely made prior to pathological examination. Usually, EATL occurs in adults and is often associated with weight loss, diarrhea, or bowel obstruction [2]. It is a rare tumor that accounts for less than 5% of all gastrointestinal lymphomas and less than 1% of all non-Hodgkin lymphomas [3]. Type 2 EATL has been renamed as monomorphic epitheliotropic intestinal T-cell lymphoma [4].

Patients typically present with abdominal pain, often associated with intestinal (jejunal) obstruction, perforation, or bleeding. Other presentations include systemic/B symptoms (fever, chills, night sweats, weight loss), fatigue, infection, hepatosplenomegaly, and pruritis [5]. The majority of patients present with Stage IV disease and bone marrow involvement is uncommon. Involvement of the lungs, skin, and soft tissue is very rare. Men are twice as likely to be affected as women, at a median age of 59 (23 - 89) years [6].

## Case presentation

The patient was a 64-year-old male with a past medical history significant for hypertension, hyperlipidemia, and diabetes mellitus type 2. He presented to the emergency room with severe abdominal pain with an episode of almost passing out at home. He was in his usual state of health and noticed intermittent abdominal pain associated with nausea and vomiting for two to three weeks. He was brought to the emergency room where a computerized tomography (CT) scan of the abdomen showed a perforation involving the distal jejunum/proximal Ileum and free air. Subsequently, he underwent exploratory laparotomy and small bowel resection.

**Figure 1:**
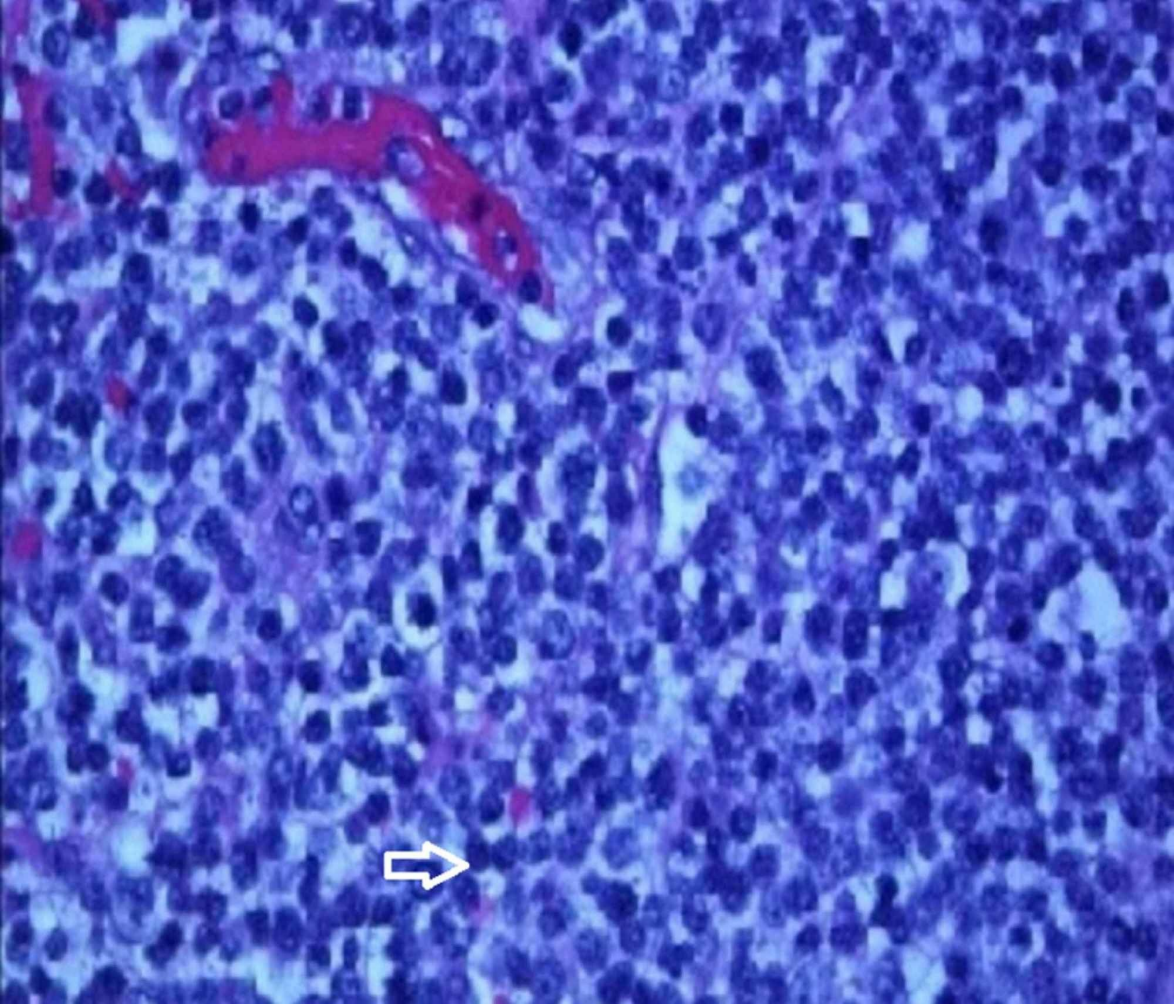
High-power histology slide of the resected specimen showing atypical lymphocytes (Arrow)

**Figure 2:**
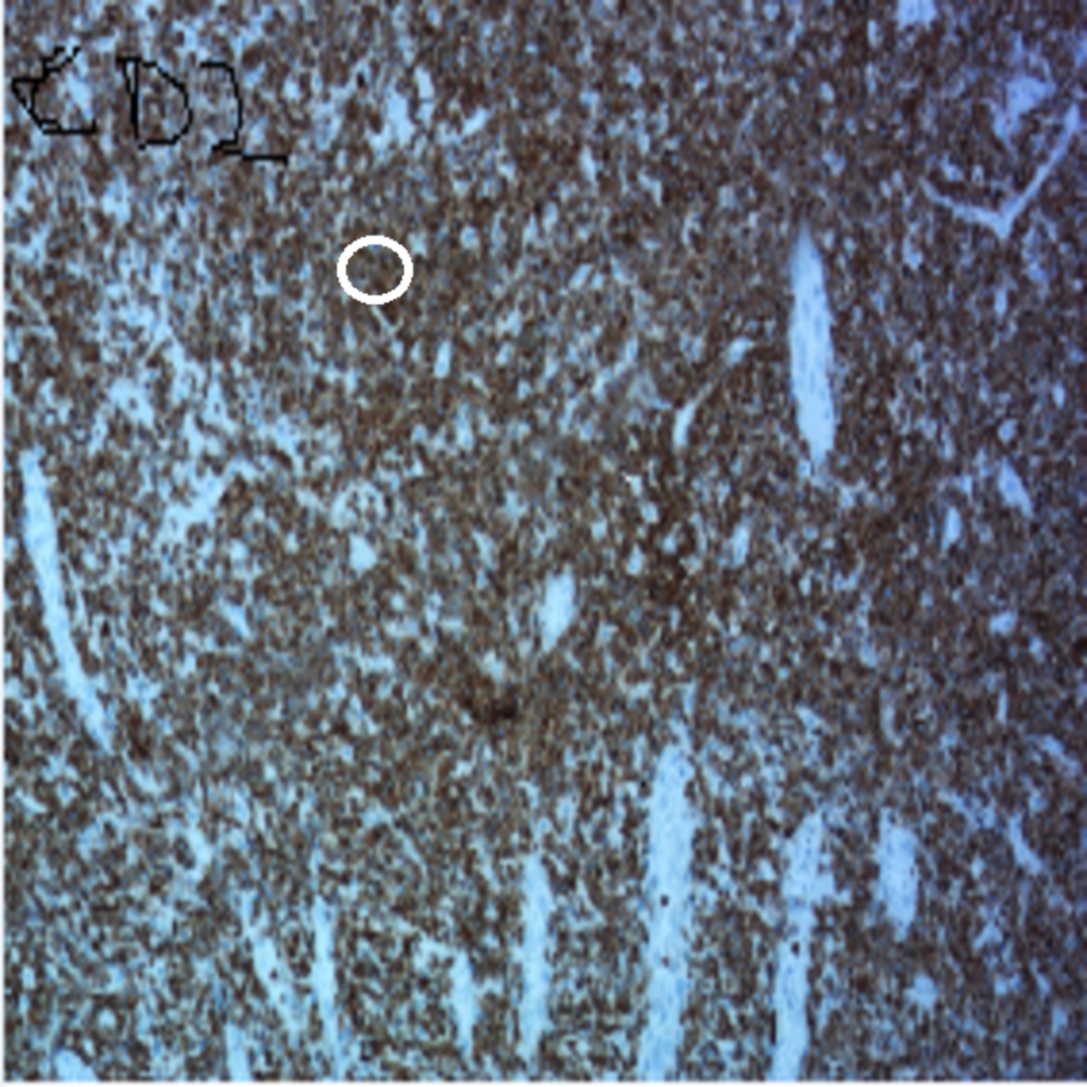
Immunohistochemical staining of the tissue showing CD2-positive tumor cells (circle)

**Figure 3:**
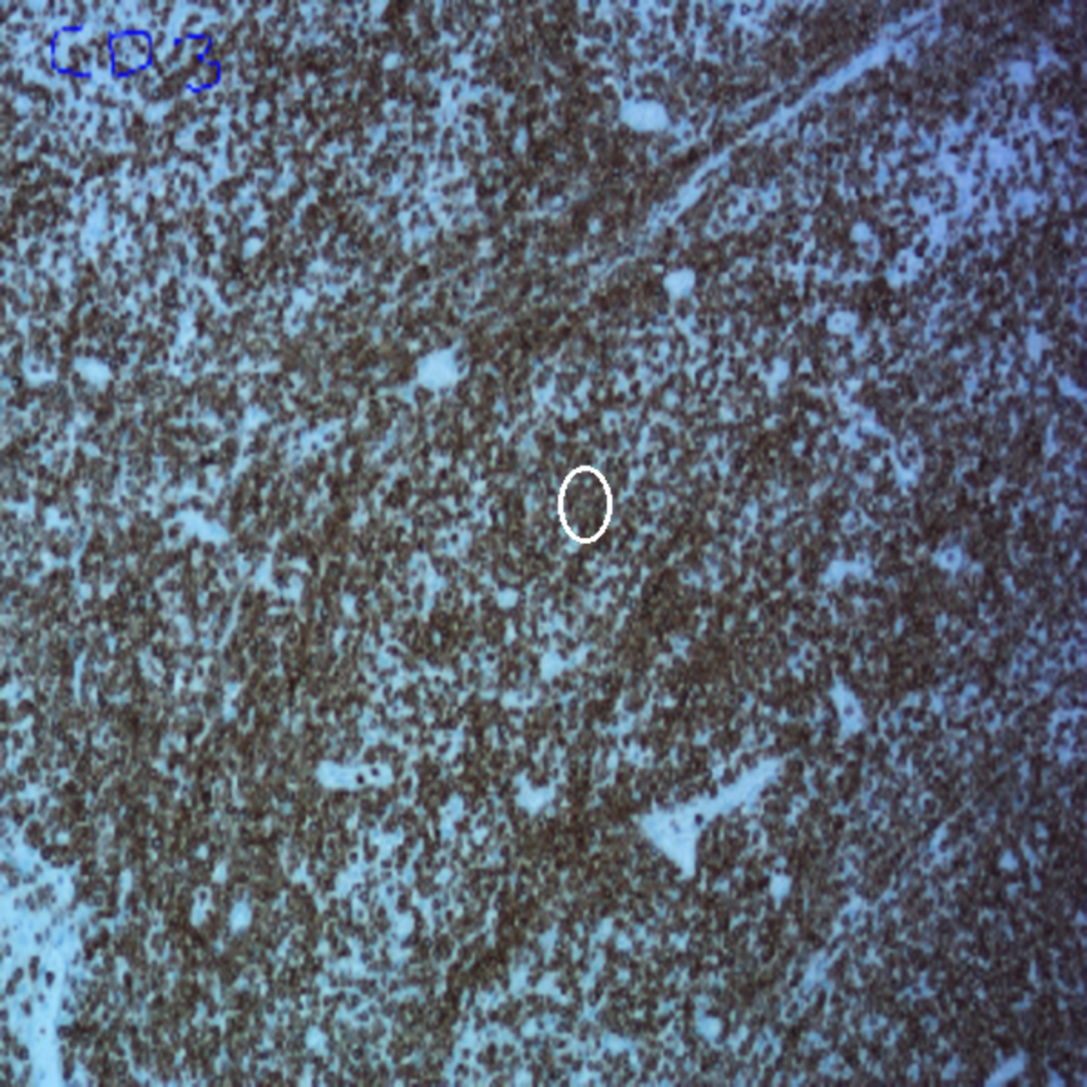
Immunohistochemical staining of the tissue showing CD3-positive tumor cells (circle)

**Figure 4:**
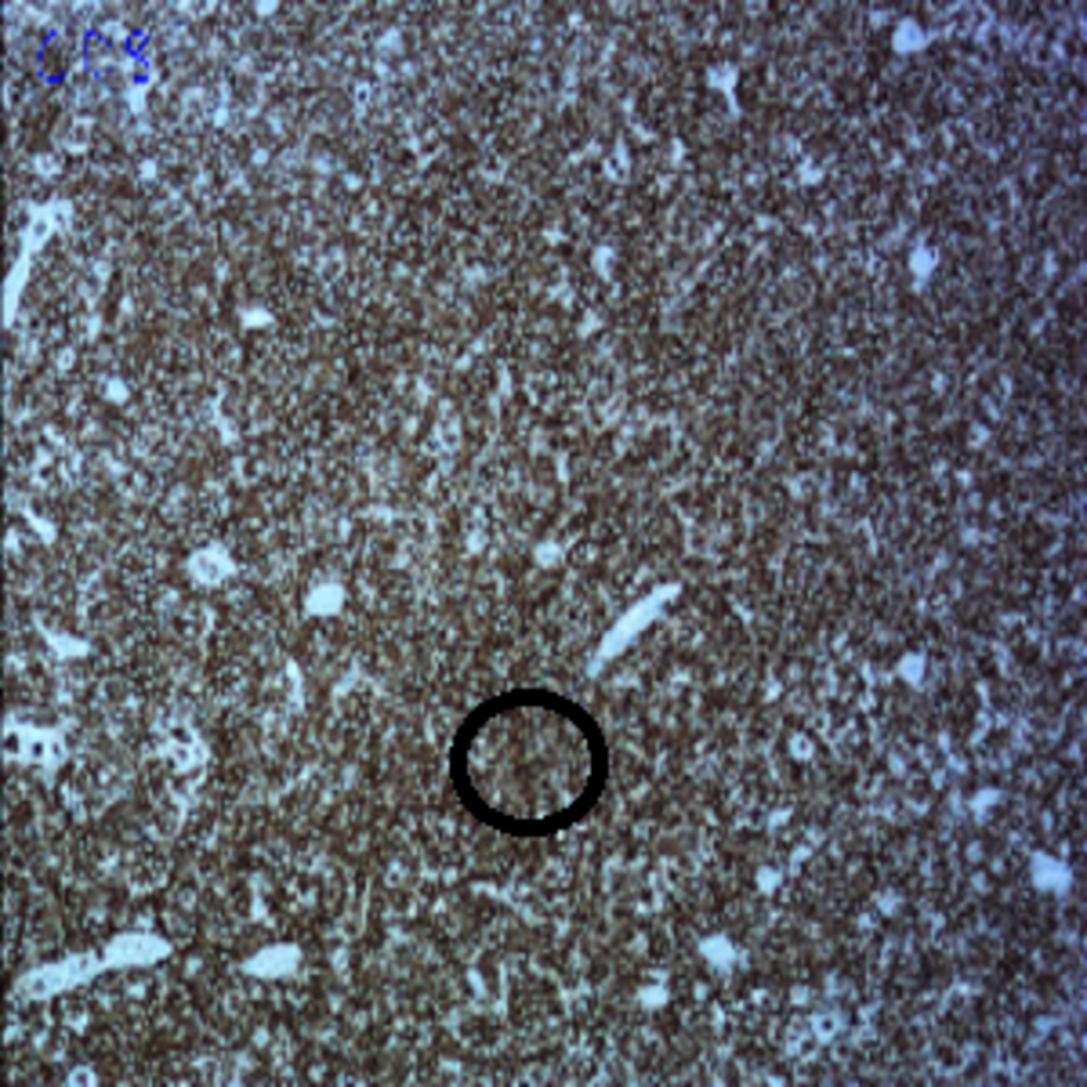
Immunohistochemical staining of the tissue showing CD8-positive tumor cells (circle)

The pathology of the resected specimen showed EATL type 2. The neoplastic cells had medium-sized round, darkly staining nuclei with a rim of pale cytoplasm (Figure 1). Intraepithelial lymphocytosis appeared to involve the adjacent intestinal mucosa. Immunohistochemistry showed the tumor cells were CD3+, CD 2+, CD8+ (Figures 2-4), CD56+, and CD 4-negative, which are markers for EATL. The Ki-67 stain, which is a marker of proliferation, showed an 80% proliferative rate. In-situ hybridization for Epstein-Barr virus‐encoded ribonucleic acid (EBER) was also performed which was negative for the Epstein-Barr virus (EBV) antigen. EBER is key to differentiate EATL type 2 from natural killer (NK)/T-cell lymphoma, which would demonstrate positivity in NK/T-cell lymphoma and be negative in EATL [6].

**Figure 5:**
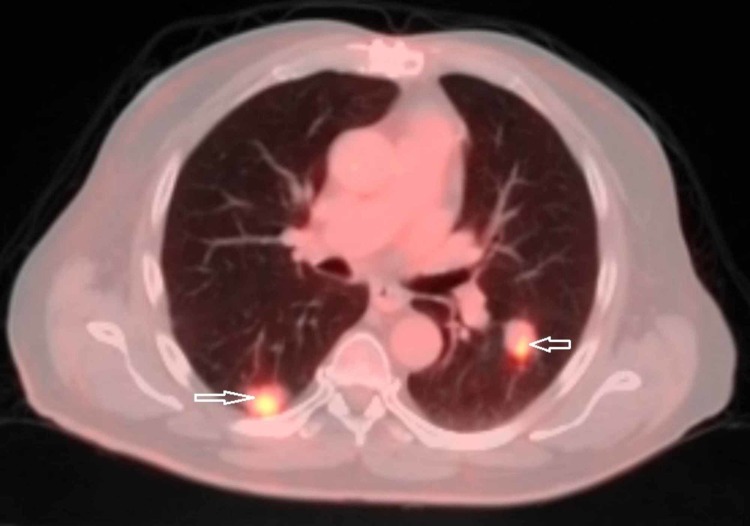
Positron emission tomography/computed tomography (PET/CT) scan showing metabolically active pulmonary nodules (arrows)

His postoperative course was complicated by a wound infection and delayed healing, leading to multiple antibiotic courses which resulted in the delay of subsequent care. A positron emission tomography/computed tomography (PET/CT) scan and bone marrow biopsy were done as an outpatient which showed multiple metabolically active pulmonary nodules. The largest nodule measured 2.6 cm in the posterior right lower lobe (Figure 5). The bone marrow biopsy revealed cellular marrow with maturing trilineage hematopoiesis and no morphologic evidence of lymphoma.

**Figure 6:**
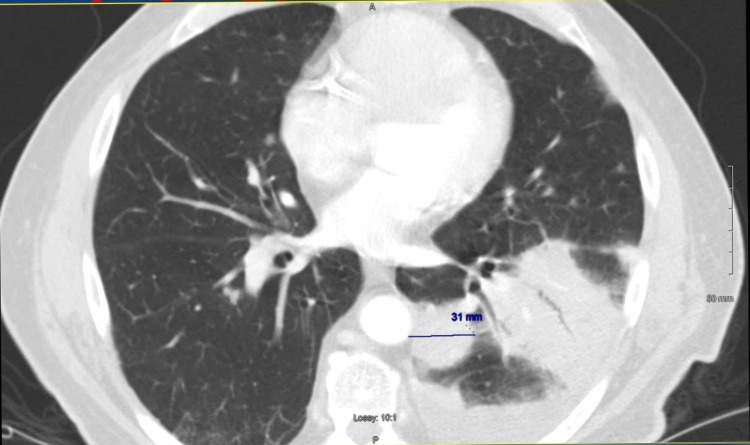
Computed tomography (CT) of the chest showing a lung mass measuring 3.1 cm

**Figure 7:**
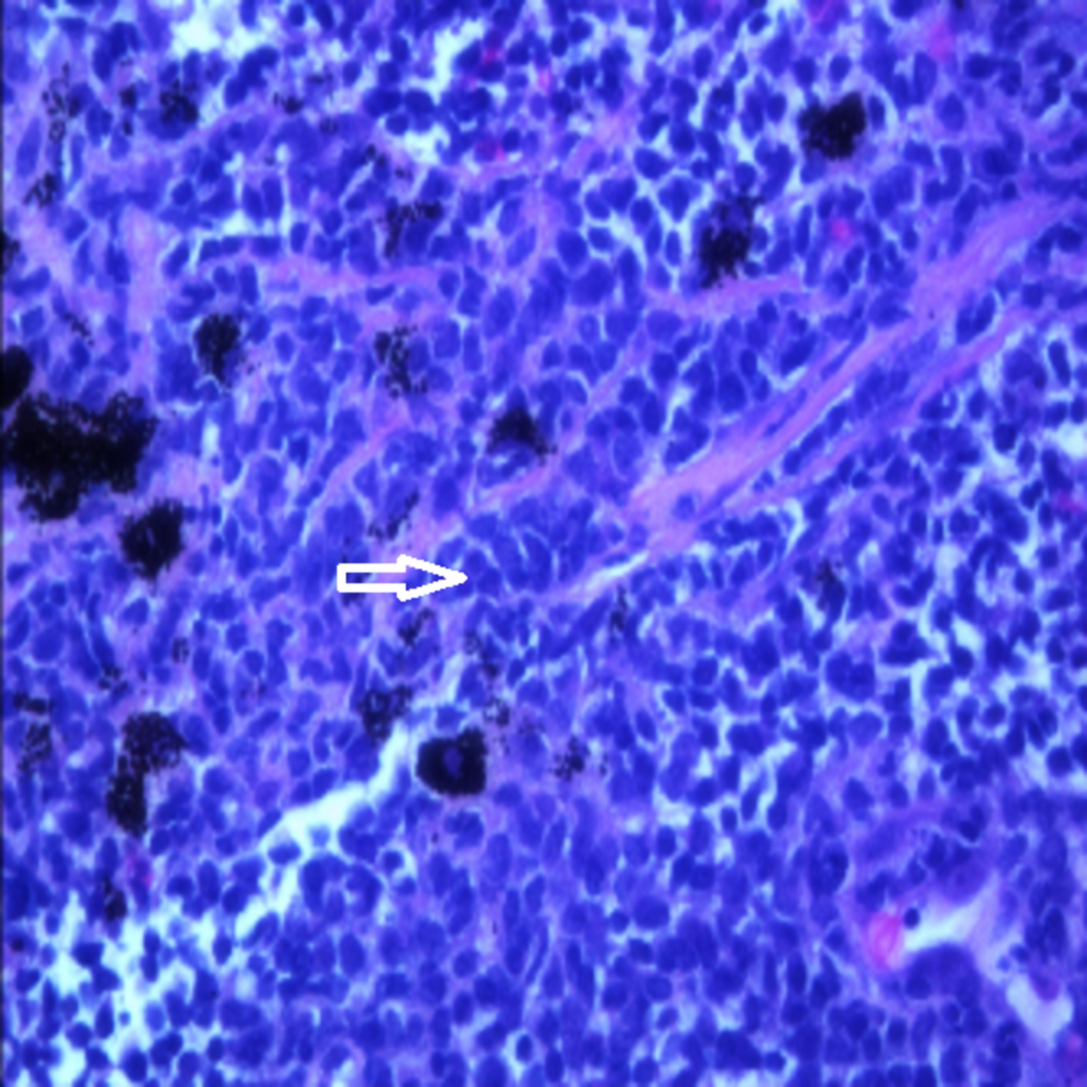
High-power slide of lung tissue showing neoplastic T-cells (arrow)

The patient subsequently presented to the hospital with shortness of breath. A CT scan of the chest showed a nodular mass-like component measuring 3.1 cm with moderate pleural effusions and multiple pulmonary nodules (Figure 6). Biopsy of the lung nodules was consistent with peripheral T-cell lymphoma consisting of small to intermediate-sized neoplastic lymphoid cells (Figure 7). The tumor cells were positive for CD3 and negative for CD20 and CAM 5.2. Ki-67 revealed a proliferative rate of 75%.

Of note, he had recurrent admissions for abdominal pain where he had small bowel perforations and had surgical resections. Subsequently, he was started on chemotherapy with the CHOP regimen (cyclophosphamide, hydroxydaunomycin, Oncovin®, prednisone) once he recovered from his surgery. After he underwent the first cycle of chemotherapy, he, unfortunately, again had a perforation of the small bowel. In view of the poor prognosis, the family chose patient comfort measures only, and he succumbed to his disease.

## Discussion

The diagnosis of the EATL is made after histopathological examination of the resected specimen since the most common presentation is a perforation. It is very difficult to diagnose the tumor on imaging studies, such as a CT scan of the abdomen, as the tumor infiltrates the wall of the intestine and does not present as a mass or growth. Even on PET scan, it is difficult to diagnose the EATL as it is more of tumor infiltration of the intestinal wall. On gross examination of the intestines, multiple circumferentially oriented ulcers are typically present and are often associated with gut perforation. There may or may not be a mass. The neoplastic cells are small to intermediate in size, with round to slightly irregular nuclei, inconspicuous nucleoli, and scant pale cytoplasm. Overall, there is a fairly monotonous appearance to the tumor cells. There is typically a sparse inflammatory background, with some cases showing scattered eosinophils.

Ideally, the treatment options include chemotherapy as induction and subsequent radiation or autologous hematopoietic cell transplantation. Our patient had surgical resection as he had presented with abdominal pain due to intestinal perforation. He was subsequently started on chemotherapy with cyclophosphamide, hydroxydaunomycin, Oncovin, prednisone which, unfortunately, he could not tolerate. 

The prognosis of EATL is very poor because of the incidence of multifocal involvement of the small intestine and frequent dissemination. Complete resection is not achievable [7]. Moreover, the high incidence of severe post-surgical complications and poor nutritional status, as was seen in our patient, can lead to progressive deterioration, preventing the use of adequate and effective treatment. Many of the patients die from complications of multifocal intestinal perforation. The median survival is usually around 10.5 months with chemotherapy and the estimated overall and failure-free survival at five years is around 20% and 4%, respectively [5], which is in line with our patient's short life expectancy following diagnosis.

## Conclusions

EATL type 2 is a rare disease with a very poor prognosis. Our patient presented with intestinal perforation and had evidence of disseminated disease. He was started on chemotherapy with a CHOP (cyclophosphamide, hydroxydaunomycin, Oncovin, prednisone) regimen; unfortunately, he had an intestinal perforation after the first cycle and subsequently passed away. Treatment in these patients is a double-edged sword, as the progression of the disease itself or chemotherapy could further thin out the intestinal wall and cause perforation. Further research needs to be done on early detection of these tumors and improved treatment options to lessen morbidity and mortality.
